# Neuroprotective effects of CD4^+^CD25^+^Foxp3^+^ regulatory T cells in a 3xTg-AD Alzheimer's disease model

**DOI:** 10.18632/oncotarget.12469

**Published:** 2016-10-04

**Authors:** Hyunjung Baek, Minsook Ye, Geun-Hyung Kang, Chanju Lee, Gihyun Lee, Da Bin Choi, Jaehoon Jung, Hyunseong Kim, Seonhwa Lee, Jin Su Kim, Hyun-ju Lee, Insop Shim, Jun-Ho Lee, Hyunsu Bae

**Affiliations:** ^1^ Department of Physiology, College of Korean Medicine, Kyung Hee University, Seoul, Republic of Korea; ^2^ K-herb Research Center, Korea Institute of Oriental Medicine, Yuseongdae-ro, Yuseong-gu, Daejeon, Republic of Korea; ^3^ Division of RI Research, Korea Institute of Radiological & Medical Sciences University of Science & Technology, Gongneug-dong, Nowon-ku, Seoul, Republic of Korea; ^4^ Acupuncture and Meridian Science Research Center, College of Korean Medical Science Graduate School, Kyung Hee University, Seoul, Republic of Korea; ^5^ Department of Biotechnology, Chonnam National University, Gwangju, Republic of Korea

**Keywords:** Alzheimer's disease, regulatory T cells (Treg), amyloid-beta (Aβ) pathology, Immunology and Microbiology Section, Immune response, Immunity

## Abstract

Alzheimer's disease patients display neuropathological lesions, including the accumulation of amyloid-beta (Aβ) peptide and neurofibrillary tangles. Although the mechanisms causing the neurodegenerative process are largely unknown, increasing evidence highlights a critical role of immunity in the pathogenesis of Alzheimer's disease. In the present study, we investigated the role of regulatory T cells (Tregs) on Alzheimer's disease progression. First, we explored the effect of Tregs (CD4^+^CD25^+^ T cells) and Teffs (CD4^+^CD25^−^ T cells) in an adoptive transfer model. Systemic transplantation of purified Tregs into 3xTg-AD mice improved cognitive function and reduced deposition of Aβ plaques. In contrast, adoptive transfer of Teffs diminished behavioral function and cytokine production. Next, we transiently depleted Treg population using an anti-CD25 antibody (PC61). Depletion of Tregs for four months resulted in a marked aggravation of the spatial learning deficits of six-month-old 3xTg-AD mice. Additionally, it resulted in decreasing glucose metabolism, as assessed by positron emission tomography (PET) with ^18^F-2 fluoro-2-deoxy-D-glucose ([F-18] FDG) neuroimaging. Importantly, the deposition of Aβ plaques and microglia/macrophage was increased in the hippocampal CA1 and CA3 regions of the Treg depleted 3xTg-AD compared to the vehicle-treated 3xTg-AD group. Our finding suggested that systemic Treg administration ameliorates disease progression and could be an effective Alzheimer's disease treatment.

## INTRODUCTION

Alzheimer's disease (AD) is the most prevalent form of dementia in the elderly individuals, characterized by the progressive deterioration of cognitive ability, accumulation of amyloid-β peptide and synaptic alterations [1]. AD accounts for over 50% of all dementia cases, and it is expected to grow dramatically, with more than 115 million people affected worldwide by 2050 [2]. AD is a neurodegenerative disorder of complex etiology that involves environmental, genetic and lifestyle risk factors. The neuropathological findings of AD are the extracellular deposition of amyloid beta peptide in the form of senile plaques and the formation of intracellular neurofibrillary tangles consist of hyperphosphorylated tau protein [3, 4].

Therapeutic approaches for Alzheimer's disease often attempt to attenuate synaptic dysfunction and improve behavior in Alzheimer's disease model [5, 6]. Amyloid-β immunotherapy is one of the most promising disease-modifying therapies for AD, which aims to reduce the amyloid burden [7, 8]. Clinical studies of Aβ-directed vaccination strategies have revealed that treatment after disease onset has little effect on the cognition of patients and has serious adverse effects [9, 10]. Despite the extensive research effort to find effective treatment for Alzheimer's patients, current therapies are no significant benefits.

CD4^+^CD25^+^Foxp3^+^ regulatory T cells (Tregs) play important roles in the maintenance of self-tolerance and the immune system [11]. Recently, modulating the function of Tregs has been proposed as a potential therapeutic option to mediate neuroprotection in neuroinflammation-mediated disorders, including Parkinson's disease and amyotrophic lateral sclerosis [12, 13]. However, studies demonstrating discrepant Treg functions in Alzheimer's disease have been reported. Baruch *et al.* showed the transient depletion of systemic Foxp3^+^ Treg-mediated immunosuppression as a negative player in AD pathology [14]. In contrast, several studies reported Treg cells as a neuroprotective immunomodulator in Alzheimer's diseases [15, 16]. Dansokho *et al*. showed that transient depletion of Treg cells accelerated the onset of cognitive deficits and was associated with modulation of the microglial response to deposits of Aβ peptide [17].

In this study, we demonstrated that Treg administration has a neuroprotective effect on Alzheimer's disease pathology and cognitive function in 3xTg-AD mice, whereas those of Teffs resulted in declined learning and memory capacities. Tregs had an impact on cognitive function, decreasing amyloid-beta deposition and inflammatory cytokine levels. Conversely, depletion of Tregs accelerated the onset of cognitive deficit, increased the amount of Aβ burden, increased microglia/macrophage responses and decreased glucose metabolism in 3xTg-AD mice. Our findings suggest that adoptive transfer of Tregs significantly protects the development of AD pathology, and depletion of Tregs by anti-CD25 antibodies has been found to mitigate disease severity

## RESULTS

### Adoptive transfer of Treg populations slowed learning and memory deficits in 3xTg-AD mice

**Figure 1:**
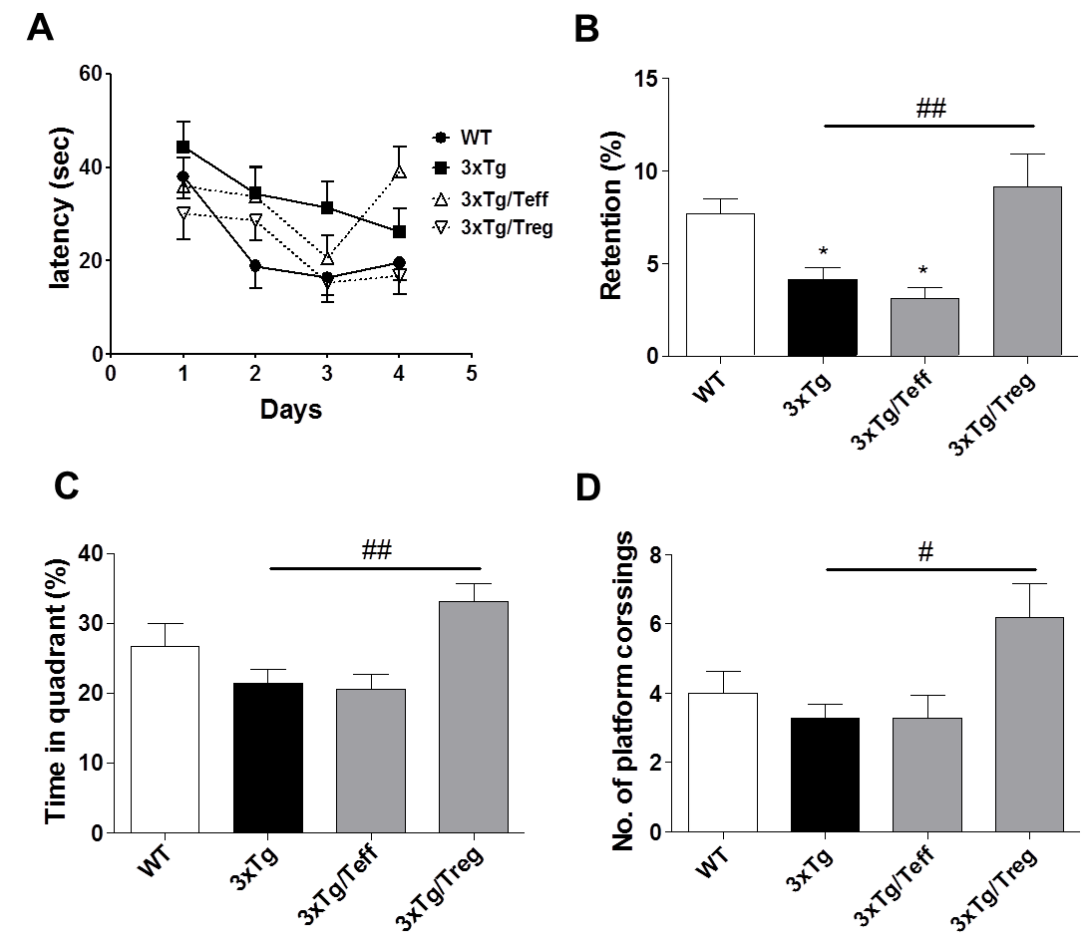
Adoptive transfer of Treg population improves spatial learning memory in 3xTg-AD mice Male 3xTg-AD mice were subjected to the Morris water maze (MWM) after two months of adoptive transfer. Training trials (60 sec each) were performed three times a day for four days for the acquisition test. In the retention test, the mice received a probe trial in which the platform was removed from the pool. Treg transferred mice showed improved spatial learning memory in the latency **A.**, retention **B.**, time in quadrant **C.** and number of platform crossing, relative to 3xTg-AD controls. The data are shown as the means ± SEM. Significance (^*^P < 0.05 *vs*. the WT group, ^#^*P* < 0.05 and ^##^*P* < 0.01 *vs*. the 3xTg group).

To measure the effect of Treg/Teff cell transfer on spatial learning and memory ability of the mice, the Morris water maze (MWM) test was conducted. 3xTg AD mice generates extracellular Aβ deposits and hyperphosphorylated tau protein in the cortex, hippocampus and amygdala in age-dependent manner [18]. Shown in the training and spatial probe test, Treg treatment on 3xTg-AD mice improved cognitive impairments compared with the 3xTg group, as shown by a decreased escape latency response and increased platform entries in the spatial probe test compared with the 3xTg group (Figure 1A and 1B). The time in target quadrant and number of platform crossing were measured during retention trials, Treg transferred mice showed a significant increase relative to the 3xTg-AD mice for this measurement (Figure 1C and 1D).

### Effects of Treg administration on inflammatory cytokine production in 3xTg-AD mice

**Figure 2:**
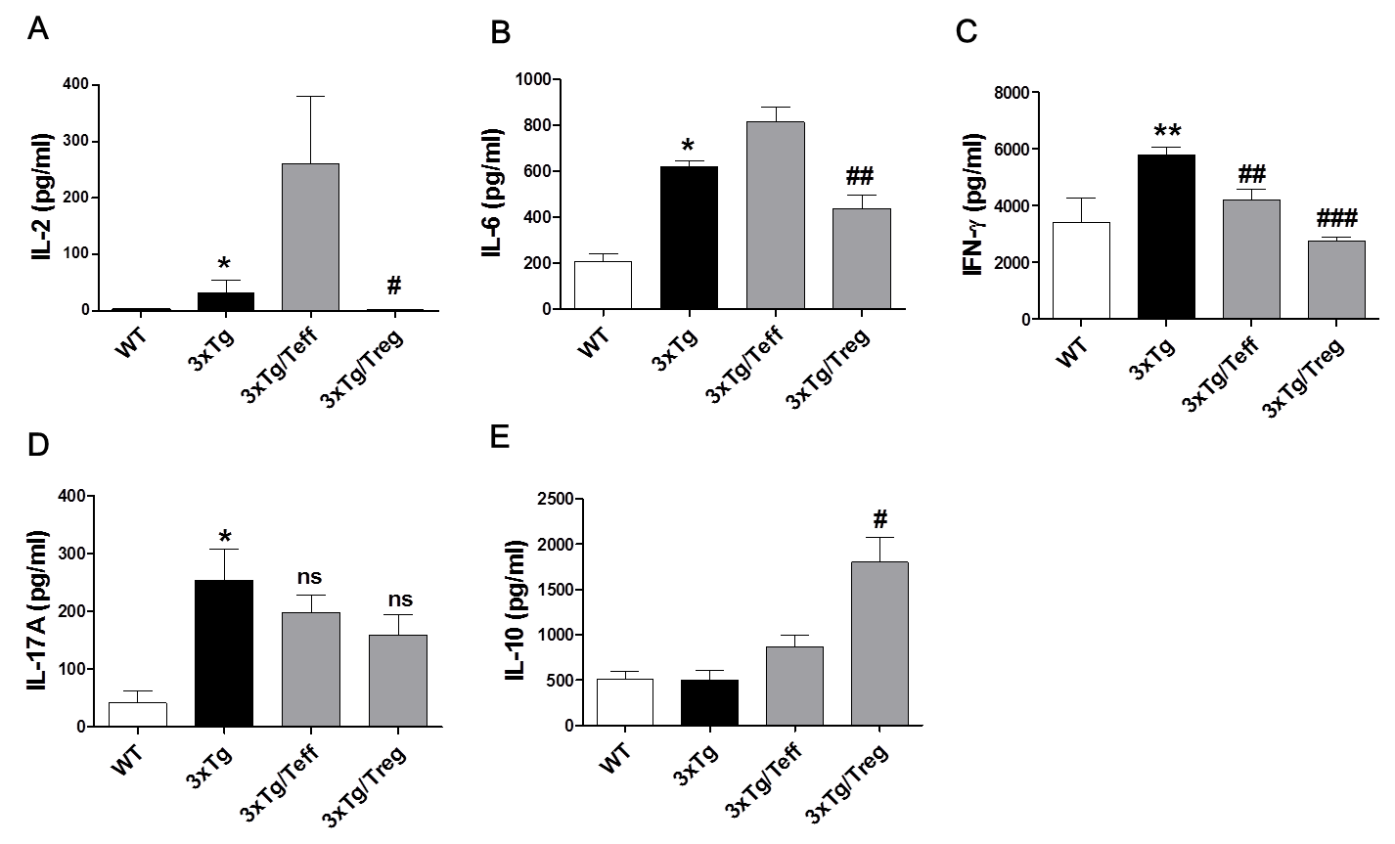
Effects of adoptive transfer on inflammatory cytokine production in 3xTg-AD mice Splenocytes were isolated from Treg or Teff cells transferred 3xTg-AD mice. Levels of I-2 **A.**, IL-6 **B.**, IFN-γ **C.**, IL-17A **D.** and IL-10 **E.** were measured using cytometric bead array (CBA) mouse Th1/2/17 cytokine kit. The data are shown as the means ± SEM. Significance (^*^P < 0.05 and ^**^P < 0.01 *vs*. the WT group, ^#^*P* < 0.05, ^##^*P* < 0.01 and ^###^*P* < 0.001 *vs*. the 3xTg group).

Cytokine production is a key pathologic event in inflammatory and anti-inflammatory responses in AD. To investigate the levels of immune response mediators, the secretion of pro-inflammatory cytokine (IL-6), Th1 cytokines (IL-2 and IFN-γ), Th17 cytokine (IL-17A) and IL-10 in supernatants of splenocyte cultures was analyzed. The expression of IL-6, IFN-γ and IL-17A was significantly increased in 3xTg-AD mice compared with the WT group (Figure 2B-2D). Administration of Tregs dramatically decreased these cytokine levels compared to the 3xTg group. Interestingly, IL-2 levels were significantly increased in splenocytes cultures of Teff cells transferred 3xTg-AD mice (the Teff group) compared with the WT or Treg group (Figure 2A). The increase observed in IL-6, IFN-γ and IL-17A levels may be related to the accumulation of intraneuronal Aβ peptides. Furthermore, IL-10 levels, a cytokine associated with Treg cells, were greatly augmented in Treg transferred 3xTg-AD mice (Figure 2E).

### Transplantation of Treg cells modulated cerebral Aβ burden and microglial activation in 3xTg-AD mice

**Figure 3:**
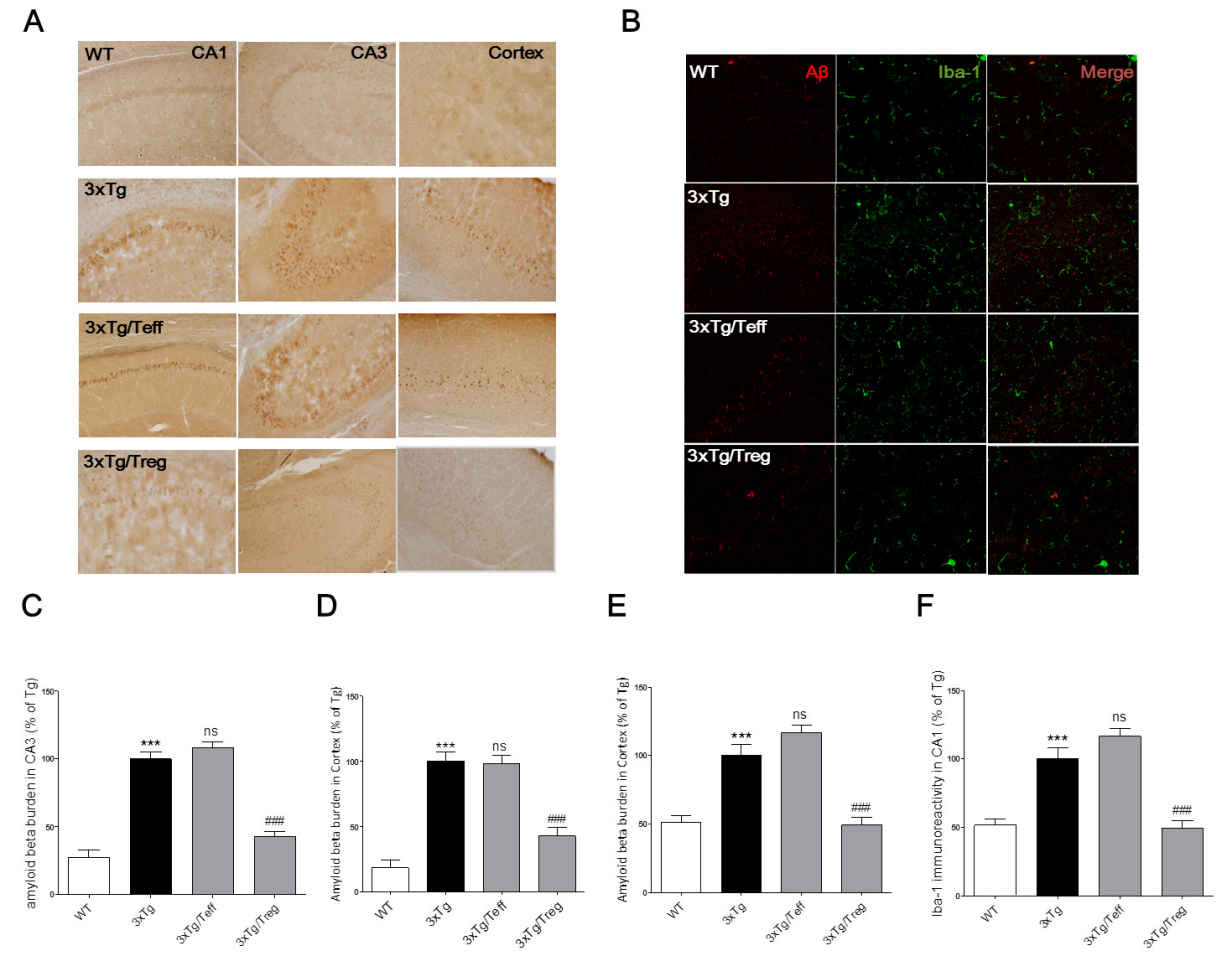
Effects of adoptive transfer on Aβ deposition and microglia activation in the brain of 3xTg-AD mice Aβ burdens were detected by immunohistochemical staining of serials brain section in cortex and hippocampus using anti-Aβ antibody (Magnification x200) **A.**. Aβ (red) and microglia (green) were detected in the hippocampal CA1 region (Magnification x400) **B.**. The percentage of Aβ burdens of brain section in hippocampal region of CA1 **C.**, CA3 **D.** and cortex **E.**. The percentage of Iba-1 positive microglia on the brain sections was analyzed **F.**. The data are shown as the means ± SEM. Significance (^***^P < 0.001 *vs*. the WT group, ns > 0.05 and ^###^*P* < 0.001 *vs*. the 3xTg group).

Changes in amyloid-beta burden were observed by immunohistochemistry in 3xTg-AD mice treated with Treg or Teff cells derived from WT mice. Figure 3 shows that treatment with Treg populations reduced Aβ burdens in both cortex and hippocampus compared with the 3xTg group. Moreover, treatment with Teff populations increased Aβ in hippocampal CA3 region in contrast to treatment with Treg populations, whereas no differences were detected in hippocampal CA1 and cortex. The response of cerebral microglia is related to the accumulation of Aβ and AD pathogenesis. Therefore, we analyzed Iba-1^+^ microglia after Treg or Teff administration. Figure 3B shows that Treg treatment reduced the number of Iba-1^+^ microglia in the brain of 3xTg-Ad mice, whereas Teff treatment increased cerebral Iba-1 expression in comparison with Treg treatment.

### Depletion of Treg cells aggravates the cognitive decline of six-month-old 3xTg-AD mice

**Figure 4:**
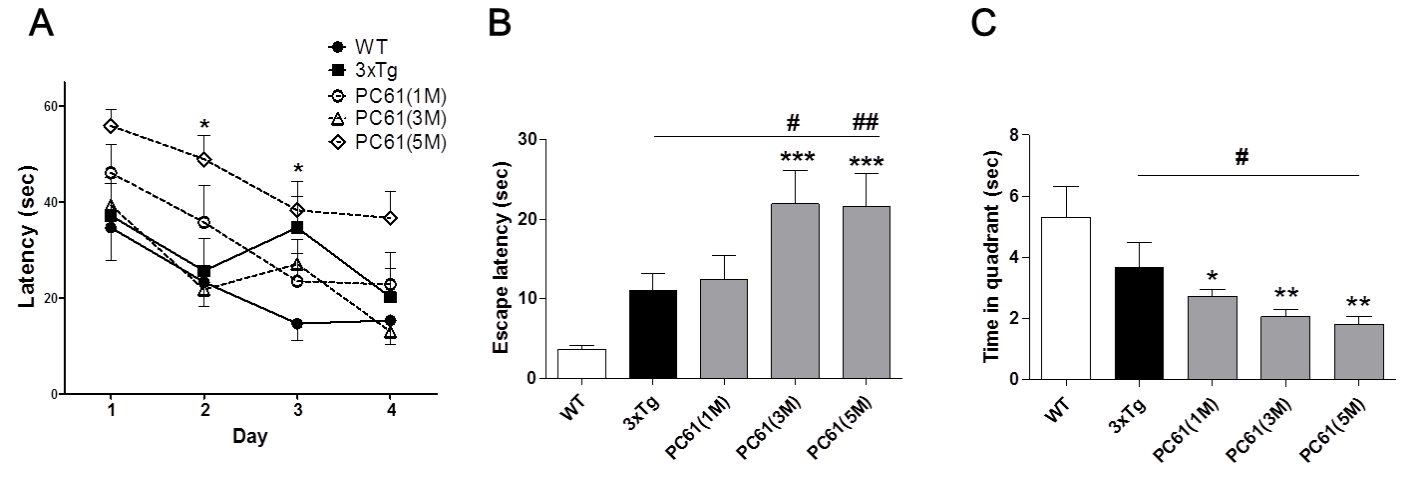
Depletion of Tregs accelerates spatial learning deficits in 3xTg-AD mice Four-week-old 3xTg-AD mice and WT littermates were injected intraperitoneally with 0.5 mg of anti-CD25 antibody (PC61) and treatment was repeated once a week for 4, 12 and 20 weeks. The Morris water maze was used to measure spatial learning memory of Treg-depleted 3xTg-AD mice. The task was performed for three trials per day over four days for the acquisition test **A.**. Escape latency of probe trials on the fifth day was measured **B.**. In the retention test, the mice received a probe trial in which the platform was removed from the pool **C.**. The data are shown as the means ± SEM. Significance (^*^P < 0.05, ^**^P < 0.01 and ^***^P < 0.001 *vs*. the WT group, ^#^*P* < 0.05 *vs*. the 3xTg group).

We depleted the Treg population of 3xTg-AD mice (four-weeks-old) by injection of 0.5 mg anti-CD25 antibody (PC61) once a week for one, three and five months. We determined the specificity and duration of PC61 antibody in spleen and lymph node on day 1, 3, 5, and 7 for CD25 depletion. Treatment of PC61 antibody eliminated CD4^+^CD25^+^ cells after 1 day of injection and the depletion was sustained for 7 days (data not shown). Furthermore, we determined whether Treg depletion from mice influences on other cell population. No changes were found in CD3, CD8, CD11c and B220 positive cell populations. We concluded that injection of anti-CD25 antibody specifically depleted CD25^+^ cell populations and the effects sustained for 7 days. After six months, we investigated whether the depletion of Tregs induces altered hippocampal-dependent spatial memory in the MWM. The latencies to find the hidden platform of the PC61 (5M) group were significantly longer than those of the 3xTg group (Figure 4A). The escape latencies on the probe trial were dramatically increased (*P* < 0.001) in the PC61 (3M) group and PC61 (5M) group, whereas 3xTg-AD control mice portrayed an inability to find the previous location of the platform (Figure 4B). The time in target quadrant during retention trials on the 5^th^ day are shown in Figure 4C. The PC61 (3M) group and PC61 (5M) group exhibited a significantly decreased time spending in the target quadrant than the 3xTg group (*P* < 0.01).

### Frequency of CD4 and CD8 lymphocytes in Treg-depleted AD mouse model

**Figure 5:**
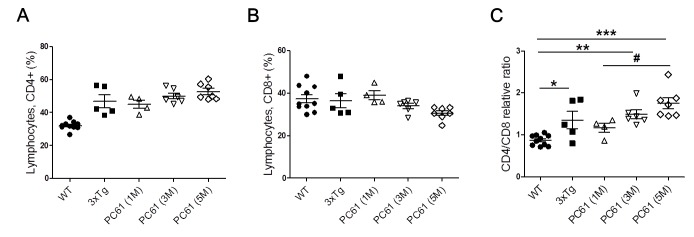
Frequency of CD4^+^ and CD8^+^ lymphocytes in spleen of Treg-depleted 3xTg AD mice Percentages of CD4^+^ **A.** and CD8^+^ T lymphocytes **B.** and CD4:CD8 ratio **C.** in WT, 3xTg and Treg-depleted 3xTg mice. The data are shown as the means ± SEM. Significance (^*^P < 0.05, ^**^P < 0.01 and ^***^P < 0.001 *vs*. the WT group, ^#^*P* < 0.05 *vs*. the 3xTg group).

We analyzed CD4 and CD8 populations in spleen of Treg-depleted AD mice compared with WT and non-depleted AD mice. In the previous reports demonstrated that increase in the populations of CD4^+^ T cells and a decrease of CD8^+^ T cells in AD patients compared to healthy controls [19, 20]. In our data, CD4^+^ T cells were slightly increased in 3xTg AD mice compared with the WT, whereas no significant difference was detected in CD8^+^ cells. As shown in Figure 5A, there is no significant decrease in the CD4^+^ T cell populations in the Treg-depleted 3xTg group compared with the 3xTg group. The increase in CD8^+^ T cells was more marked for the PC61 (5M) group compared to the 3xTg AD mice, but this difference also did not reach significant level (Figure 5B). The CD4/CD8 ratios in the Treg-depleted mice were higher than the ratio in the 3xTg AD mice (Figure 5C).

### Effects of CD25 depletion on amyloid-beta pathology and neuroinflammatory responses in the 3xTg AD mice

**Figure 6:**
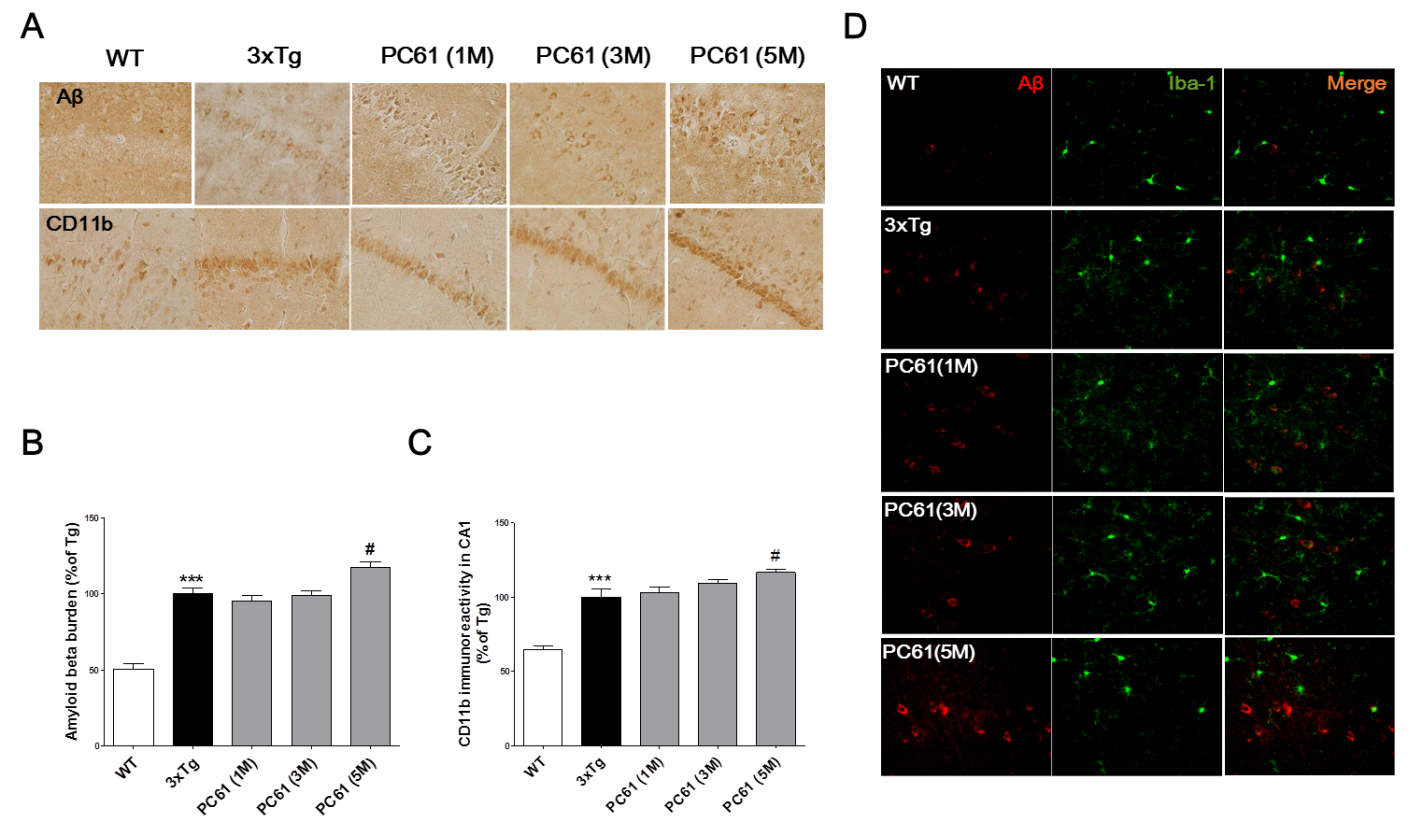
Depletion of Tregs aggravates Aβ pathology and microglial deactivation in the hippocampal region of 3xTg-AD mice Aβ deposits were detected with immunohistochemical staining with anti-Aβ antibody in the hippocampal CA1 region. CD11b positive microglia was detected in the hippocampal CA1 region. The data are shown as the means ± SEM. Significance (^***^P < 0.001 *vs*. the WT group, ^#^*P* < 0.05 *vs*. the 3xTg group) (Magnification x 400).

Amyloid-beta deposits are observed in the hippocampus from six month old 3xTg AD mice [21, 22]. Amyloid-beta burden was significantly increased in the hippocampal CA1 region of six month old 3xTg AD mice compared to the age matched WT (*P* < 0.001, Figure 6A and 6B). Compared to the 3xTg AD mice, the amyloid-beta burden in CA1 regions was significantly increased in PC61 (5M) group (117.1 ± 3.68%, *P* < 0.05). But, there were no significant differences in one month or three month treatment of PC61 (1M, 3M) (Figure 6A and 6B). To investigate whether the depletion of CD25 resulted in the increase of microglial activation,, immunohistochemistry using anti-CD11b antibody was performed in the hippocampus. In the WT mice, only a few CD11b positive microglia were observed in the brain sections (Figure 6A and 6C). In contrast, numerous CD11b positive microglia were observed in the hippocampus of the 3xTg AD mice. The CA1 regions of PC61 (5M) groups exhibited significant increases in the amount of CD11b positive microglia, and there were no significant differences between the PC61 (1M), PC61 (3M) and the vehicle-treated 3xTg AD mice. Figure 6D shows that Treg-depletion induced the number of cerebral Iba-1^+^ microglia in the brain of 3xTg AD mice.

### Changes in brain glucose metabolism

**Figure 7:**
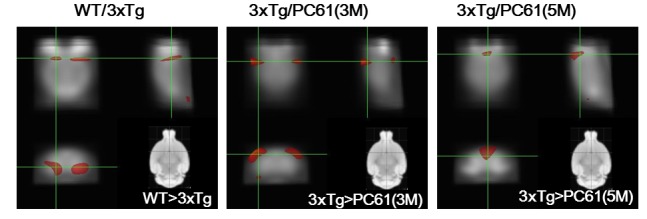
Depletion of Tregs decreases glucose uptake in the brain Voxel-wise comparisons between the WT and 3xTg, 3xTg and PC61 (3M), and 3xTg and PC61 (5M) group. The brain regions in which regional FDG uptake in the WT group were significantly greater than those in the 3xTg group. The brain regions in which regional FDG uptake in the 3xTg group were significantly greater than those in the PC61 (3M and 5M) group. Significance (*P* < 0.05).

We determined that the effect of CD25 depletion corresponds to enhancing the brain glucose uptake/metabolism using FDG-PET imaging. The result showed that the uptake of ^18^F-FDG in hippocampus of the WT was significantly increased comparing with3xTg AD mice (*P* < 0.005) (Figure 7). No significant difference in glucose uptake between the 3xTg and the PC61 (1M) group was found. The uptake of 18F-FDG in the cortex of 3xTg AD mice was significantly increased compared to three month treatment of PC61 (3M) group. The glucose metabolism of the 3xTg group in the temporal lobe was remarkably increased when compared with five month treatment of PC61 (5M) group.

## DISCUSSION

In this study, we transplanted Treg cells into 3xTg-AD transgenic mice to ameliorate Alzheimer-like pathology. Our results showed that Treg cells not only improved spatial learning and memory of 3xTg-AD mice but also contributed to the reduced cerebral Aβ burden and decreased inflammatory cytokine productions. Alternatively, Teff cells worsened cognitive deficits and increased inflammatory cytokines. Although Teff treatment did not significantly reduce cerebral Aβ burden compared with the 3xTg group, there was an obvious decrease in Aβ burden in the Treg group compared with the Teff treatment. Furthermore, Treg and Teff treatments altered the microglia response in brains of 3xTg-AD mice.

Microglial cells are the primary immune effector cells in the central nervous system (CNS) and play an important role in the tissue maintenance and immune surveillance [23]. Under pathological situation, microglial cells become activated, migrate and secrete inflammatory mediators for neural cell plasticity and survival. In murine models of AD, amyloid plaques form in the brain, followed by the appearance of microglia that become associated with the amyloid plaque [24]. Elevated levels of a number of microglial-derived cytokines and other immune mediators were found in the brain of AD patients. We showed that activated microglia cells were increased in the brain of 3xTg-AD mice compared with WT mice. Microglial cells were significantly reduced by Treg administration, whereas no significant differences were found between the 3xTg and 3xTg/Teff group. Reduction of microglia responses and protection against Alzheimer's disease pathology suggests the possible involvement of Treg cells in inhibiting inappropriate or excessive immune responses.

Cerebral glucose metabolic activity is a hallmark of synaptic function and density [25]. Decline of synaptic density in the brain is a common feature of neurodegenerative disorders and strongly correlates with the cognitive decline observed in AD. ^18^F-2-fluoro-deoxy-D-glucose positron emission tomography (^18^F-FDG-PET) is used to evaluate regional cerebral metabolic rate for glucose information. ^18^F-FDG-PET is considered a neuroimaging biomarker with a highly sensitive indicator for the diagnosis of AD [26]. Decreased ^18^F-FDG uptake represents a reduction in neuronal energy demand, mainly arising from synaptic loss caused by Aβ and tau pathology in AD patients. Here, we identified the pattern of regional cerebral metabolic changes after depletion of Treg cells in 3xTg-AD mice. The cerebral glucose metabolism was compared between each group by a voxel-based method of analysis. Compared with the control group (WT), the 3xTg-AD group exhibited lower levels of glucose metabolism. Depletion of Tregs for three and five months significantly reduced the level of glucose uptake, whereas no differences were shown in comparison of the 3xTg and PC61 (1M) group. Consistent with our study, several reports have found reduced cerebral glucose metabolism in patients with AD [27, 28].

The involvement of Treg cells in AD is not fully understood. The analysis of Treg from AD patients revealed that the frequency of Treg increases with age is accompanied by higher suppressive activity [29]. Saresella *et al*. found that PD1^+^ Tregs are increased in both patients with fully-developed AD or with mild cognitive impairment compared with healthy controls [30]. In contract with our result, Baruch *et al*., reported that transient depletion of Tregs or pharmacological inhibition of Treg activity was shown decreased Aβ clearance [14]. They used cross-bred 5XFAD AD-Tg mice with Foxp3-DTR mice for transient depletion. However, the major differences are the stage and duration of the Treg depletion. We started Treg depletion in an early stage of Alzheimer-like pathology, 4 weeks of age in 3xTg-AD model, for 4, 12 and 20 weeks, whereas Baruch *et al*., transiently depleted Tregs at an intermediated stage. Although dysfunctions of Tregs occurring only in the early stages of AD development cannot be fully uncovered, several studies show Tregs as a contributor to the pathogenesis of AD. A recent study showed that systemic transplantation of autologous Tregs from AβPPswe/PS1dE9 double-transgenic mice after human umbilical cords-derived mesenchymal stem cells (hUC-MSCs) education attenuated the cognitive deficit, amyloid-beta deposits and microglial activation in Alzheimer's disease mice [31]. TGF-β1, mainly secreted by Treg cells, was found to possess anti-neuroinflammation activity in Aβ_1-42_ induced AD model [32]. These findings emphasized the importance of Tregs in neurodegeneration.

In summary, the findings in the present study show that Treg cells play a beneficial role in the pathophysiology of AD and attenuate disease progression in a murine model of AD. Treg administration was shown to reduce AD development in 3xTg-AD mice, whereas Teff administration increased cognitive deficits and boosted inflammatory cytokines. Depletion of Treg cells accelerated the cognitive deficit, increased Aβ deposition and reduced glucose metabolism in the brain. Taken together, Treg cells induced immunosuppressive activity that is important for the development and maintenance of AD pathology.

## MATERIALS AND METHODS

### Animals

The 3xTg-AD mice harboring a mutant APP (KM670/671NL), which is a human mutant PS1 (M146V) knock-in, and tau (P301L) transgenes (B6;129-*Psen1*^tm1Mpm^ Tg(APPSwe, tauP301L)1Lfa/J) were obtained from Jackson Laboratory [18]. Male C57BL/6 mice were purchased from Charles River Korea (OrientBio, Sungnam, Korea). All mice were maintained under pathogen-free conditions with air conditioning and a 12-h light/dark cycle. All of the mice had free access to food and water during the experiments. The study was conducted in accordance with the Rules for Animal Care and the Guiding Principles for Animal Experiment Using Animals and was approved by the University of Kyung Hee Animal Care and Use Committee (KHUASP(SE)-13-015).

### Treg and Teff cell preparation and adoptive transfer

CD4^+^CD25^+^ T cells (Tregs) and CD4^+^CD25^−^ T cells (Teffs) were isolated from the spleens obtained from male C57BL/6 mice (six-week-old) using magnetic-activated cell sorting (MACS) according to manufacturer protocols (CD4^+^CD25^+^ Regulatory T Cell Isolation Kit; Miltenyi Biotec Inc, Auburn, CA, USA). Briefly, CD4^+^ T cells were negatively selected using a biotinylated antibody cocktail and anti-biotin microbeads and then separated into CD4^+^CD25^−^ and CD4^+^CD25^+^ T cells. CD4^+^CD25^−^ T cells were negatively selected from CD4^+^CD25^+^ Treg cells using phycoerythrin (PE)-labeled anti-CD25 antibody and anti-PE microbeads. The purity of both populations was more than 85%, as determined by flow cytometry analysis. Either 1x10^6^ Treg or Teff cells were adoptively transferred by intravenous injection into the tail of four-month-old male 3xTg-AD mice.

### Behavioral test

Spatial learning and memory in mice was examined using the Morris water maze with minor modifications [33]. Briefly, mice were trained in a circular water maze of 90-cm diameter (opaque water, 22±2°C, 6-cm platform 1 cm below water, maximal trial duration 60 s and 30 s on platform at the end of each trial). Each animal was trained for one of the different starting positions and the swimming path once per day for four days, with a new platform location each day. All mice were subjected to three trials per day at intervals of 15 min for four consecutive days. For the probe trial, the platform was removed from the pool and mice were allowed to swim freely for 60 s to search for the previous location of the platform. Data were collected using a video camera connected to a video recorder and a tracking device (S-MART, Pan-Lab, Spain)

### Measurement of cytokines in the culture of splenocytes

Single-cell suspension of splenocytes was placed in 96-well tissue culture plates in RPMI-1640 (WelGENE INC., Taegu, Korea) supplemented with 10% fetal bovine serum (FBS), 50 IU/ml penicillin and 50 μg/ml streptomycin (Hyclone, Logan, UT, USA). Cultures were activated in the presence of plate-bound anti-CD3 and soluble anti-CD28 antibody (BD Biosciences, San Jose, CA, USA). Cytokines were detected in the supernatants by a mouse cytometric bead array (CBA) kit (BD Biosciences) according to the instruction manual. Briefly, samples were added to a mixture of capture antibody bead reagent and PE-conjugated detection antibody. The mixture was incubated at room temperature in the dark and then washed. Data were acquired by BD FACS Calibur flow cytometer and analyzed using BD CellQuest, BD CBA Software (BD Biosciences).

### Tissue preparation and immunohistochemical staining

After the behavioral test, mice were transcardially perfused with saline solution containing 0.5% sodium nitrate and heparin (10 U/ml) and then fixed with 4% paraformaldehyde (PFA) in 0.1 M phosphate buffer (PB). Each brain was dissected from the skull, post-fixed overnight at 4°C, stored in a 30% sucrose solution until it sank and frozen-sectioned on a sliding microtome into 30 μm thick coronal sections. The tissue sections in citric acid buffer solution (pH 6.0) were placed in the microwave for antigen retrieval. Next, sections were put into 3% hydrogen peroxide solution, incubated at RT for 10 min to reduce endogenous peroxidase activity and washed with PBS. Amyloid-β burdens were incubated with mouse monoclonal 4G8 antibody (BioLegend, San Diego, CA, USA) overnight at 4°C. The brain sections were washed with PBS, incubated with the appropriate biotinylated secondary antibody and processed with an avidin-biotin complex kit (Vectastain ABC kit; Vector Laboratories, Burlingame, CA, USA). The bound anti-serum was visualized by incubating with 0.05% diaminobenzidine-HCl (DAB) and 0.003% hydrogen peroxide in 0.1 M PB. The DAB reaction was stopped by rinsing the tissue with 0.1 M PB. The labeled tissue sections were then mounted and analyzed under a bright-field microscope (Nikon, Tokyo, Japan). For immunofluorescence staining, fluorescent anti-mouse IgG and fluorescent anti-rabbit IgG were used for the detection of amyloid-beta and Iba-1 antibody, respectively. The slides were exposed to fluorescein isothiocyanate (FITC)- and PE-labeled secondary antibody. Fluorescent images were captured on a Zeiss LSM5 confocal microscope (Zeiss, Axioskop, Oberkochen, Germany).

### Depletion of CD4^+^CD25^+^ regulatory T cells *in vivo*

Anti-mouse CD25 rat IgG1 (anti-CD25; clone PC61) was generated in-house from hybridomas obtained from the American Type Culture Collection. The mice (four weeks old) were randomized into four groups as follows: (1) (WT) group, no treatment; (2) PC61 (1M) group, intraperitoneal (i.p.) injection with 0.5 mg of PC61 in 200 μl PBS once a week the four weeks; (3) PC61 (3M) group, i.p. injection with 0.5 mg of PC61 in 200 μl PBS once a week for 12 weeks; and (4) PC61 (5M), i.p. injection with 0.5 mg of PC61 in 200 μl PBS once a week for 20 weeks.

### [^18^F]- FDG Micro-PET scan

All of the mice fasted for 8 h before the experiments to increase the [^18^F]-fluorodeoxyglucose (FDG) uptake in the brain [34]. Before the [^18^F]-FDG uptake, all mice were warmed using a heating pad in a cage according to the optimized [^18^F]-FDG uptake protocol [35]. 1mCi of [^18^F]-FDG was injected through a tail vein, and the mice were anesthetized with 2% isoflurane in 100% oxygen (Forane solution; ChoongWae Pharma, Seoul, Korea). Siemens Inveon PET scanner (Siemens Medical Solutions, Malvern, PA, USA) was used for the PET imaging. The transverse resolution was used was < 1.8 mm at the center [36, 37]. After 10 min of uptake time, 30 min of emission PET data were acquired with350-650 keV energy window. The list-mode PET data were reconstructed using 3 DRP methods. The pixel size of the reconstructed images was 0.15 × 0.15 × 0.79 mm^3^. Attenuation, scatter corrections, and normalization were performed.

### Voxel-based statistical analysis

Voxel-based statistical analysis was performed to identify the difference of the cerebral glucose metabolisms within groups. Optimized SPM analysis, developed by one of authors in our present study, was used [38]. Briefly, the brain region was extracted using rectangular masking method. A study-specific template was constructed for spatial normalization. The PET data were then spatially normalized onto a mouse brain template and smoothed using a 3 mm Gaussian kernel. Count normalization was also performed. A voxel-wise *t*-test between the groups' datasets was performed using the SPM 8 (uncorrected *P* < 0.005).

### Statistical analysis

To determine statistical significance of more than two groups, values were compared using one-way NOVA followed by the Tukey's multiple comparison tests using Prism 5.01 software (GraphPad Software Inc., San Diego, CA, USA). Differences with a *P*-value of ≤ 0.05 were considered significant.
